# Skin Phototype Classification with Machine Learning Based on Broadband Optical Measurements

**DOI:** 10.3390/s24227397

**Published:** 2024-11-20

**Authors:** Xun Yu, Keat Ghee Ong, Michael Aaron McGeehan

**Affiliations:** 1 Department of Bioengineering, Phil and Penny Knight Campus for Accelerating Scientific Impact, University of Oregon, Eugene, OR 97403, USA; xunyu@uoregon.edu (X.Y.); kgong@uoregon.edu (K.G.O.); 2 Penderia Technologies Inc., Eugene, OR 97403, USA

**Keywords:** Fitzpatrick skin type, K-means clustering, machine learning, skin optical properties, dermatology, skin type classification

## Abstract

The Fitzpatrick Skin Phototype Classification (FSPC) scale is widely used to categorize skin types but has limitations such as the underrepresentation of darker skin phototypes, low classification resolution, and subjectivity. These limitations may contribute to dermatological care disparities in patients with darker skin phototypes, including the misdiagnosis of wound healing progression and escalated dermatological disease severity. This study introduces (1) an optical sensor measuring reflected light across 410–940 nm, (2) an unsupervised K-means algorithm for skin phototype classification using broadband optical data, and (3) methods to optimize classification across the Near-ultraviolet-A, Visible, and Near-infrared spectra. The differentiation capability of the algorithm was compared to human assessment based on FSPC in a diverse participant population (*n* = 30) spanning an even distribution of the full FSPC scale. The FSPC assessment distinguished between light and dark skin phototypes (e.g., FSPC I vs. VI) at 560, 585, and 645 nm but struggled with more similar phototypes (e.g., I vs. II). The K-means algorithm demonstrated stronger differentiation across a broader range of wavelengths, resulting in better classification resolution and supporting its use as a quantifiable and reproducible method for skin type classification. We also demonstrate the optimization of this method for specific bandwidths of interest and their associated clinical implications.

## 1. Introduction

**Figure 1:**
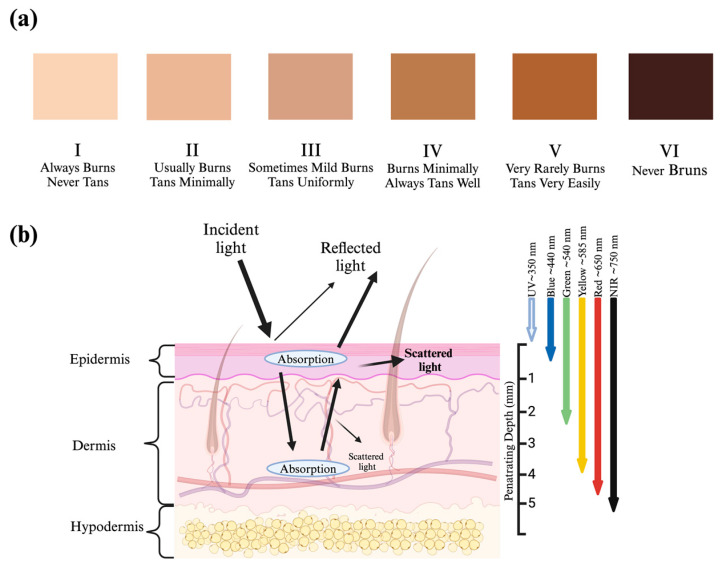
(**a**) Fitzpatrick Skin Type Scale (I–VI) and (**b**) Generalized penetration depths of various wavelengths of light through tissue structures of interest [11].

Visual skin phototype evaluations are widely used in medicine. From clinical uses such as screening for skin cancer and skin grafting immunosuppression to cultural research in colorism and cosmetic product development [1,2,3,4,5], skin phototype classification continuously demonstrates usefulness across academic, clinical, and industrial applications [6]. In addition to these use cases, skin phototype classification enables clinicians to assess tissue health to diagnose and/or treat medical conditions or risk thereof [3]. The Fitzpatrick Skin Phototype Classification (FSPC) scale (Figure 1a) was developed in 1975 to classify different skin phototypes according to their susceptibility to sunburn and skin cancer in response to ultraviolet (UV) radiation [7]. Despite its broad adoption for clinical use, the FSPC has become controversial in recent years for its underrepresentation of populations with darker skin pigmentation, poor classification resolution (i.e., limited to only six possible phototypes), and subjective nature rather than quantifying skin type through empirical evidence or measurement tools [8]. The FSPC scale also has a major limitation in its reliance on human visual data extracted from the relatively narrow visible light (VL) spectrum as inputs, which may not represent skin optical responses to UV radiation as intended. Similarly, the FSPC has limited use for clinical conditions that are not UV-sensitive, such as skin optical properties in the infrared and near-infrared spectra, which may hold significance for classifying skin phototypes for other clinical applications (e.g., personalized and optimized exposure dosage and wavelengths for photobiomodulation therapy) [9]. Furthermore, the FSPC is sometimes misused to categorize race and ethnicity rather than its intended use with UV exposure risks [10]. Each of these compounding factors limits the clinical usefulness of the FSPC and highlights a need for a quantitative and repeatable skin type classification system that can probe skin optical properties at specific optical bandwidths of interest to target specific clinical uses.

The limitations of visual chromatic skin evaluations, such as the FSPC, may have detrimental impacts on patient care, such as misleading clinicians, causing delay or deviation from optimal treatment procedures [12]. For example, inaccurate blood oxygenation measurements have been documented among patients with darker skin types compared to those with lighter skin types, increasing the possibility of undiagnosed hypoxemia [12,13,14]. Specifically, one meta-analysis reported a mean overestimation of 1.11% and 1.52% of arterial and peripheral blood oxygenation in people with high levels of skin pigmentation [12]. Moreover, patients with highly pigmented skin are subject to higher risks of undiagnosed bacterial-laden wounds based on the FSPC evaluation, leaving darker skin phototype patients at higher risk for disease progression [15]. In a review of racial disparities in dermatology, patients with dark skin phototypes were less likely to receive a variety of pharmaceutical interventions needed for dermatological care and, thus, more likely to have more severe dermatological disease progression [16]. Finally, questionnaire-based skin type classifications, such as the FSPC, are shown to overestimate pigmentation among subjects with light skin and underestimate pigment for those with dark skin, highlighting a need for a more representative skin phototype scale with more quantitative and reproducible classificational approaches based on skin optical properties instead of appearances [17,18].

Past research efforts have explored different sensor-based methods, such as colorimeters and spectrophotometers, in combination with algorithms such as the Individual Typology Angle (ITA) [19] and International Commission on Illumination L*a*b* color space (CIELAB) [20,21], or surveys to quantify and classify human skin types [22]. These approaches use one or multiple light sources to emit standardized or distributed wavelengths, allowing photodetectors to measure light reflected or transmitted through tissue [23]. Light intensity data are then used as algorithm inputs to assess skin optical characteristics. In the 3-dimensional CIE L*a*b* model, L* represents luminescence, a* measures red/green balance, and b* measures yellow/blue balance [23]. The ITA algorithm similarly models luminance, yellow, and blue light intensities as an arc, classifying skin phototypes based on angle ranges. While CIE L*a*b* and ITA offer more repeatable and quantitative measurements than visual assessments (e.g., the FSPC Scale), they are limited by narrow visible bandwidths and cannot be used to analyze high-dimensional optical data. This limits their ability to distinguish between spectral features with similar appearances [23]. Additionally, these algorithms rely on wavelength intensity ratios rather than raw irradiance values, potentially reducing sensitivity to specific physiological features and differentiation between skin types.

Different wavelengths of the incident light penetrate different tissue depths and structures (Figure 1b) [24]. For example, longer visible wavelengths, such as red light (~650 nm), penetrate deeper than shorter wavelengths, such as blue light (~450 nm). Thus, optical resonance at these wavelengths represent different anatomical and physiological features of the tissue [11,24]. Previous research investigating tissue penetration depths using different wavelengths via computational simulations [24,25] and observations from photobiomodulation Therapy (PBMT) [26] suggests that nonuniformity in tissue structures, thickness, and optical properties (i.e., absorption and scattering coefficients) [27] all contribute to difficulties in creating standardized qualitative or quantitative tools to classify skin types. Machine learning algorithms, however, show promise for aiding in skin type classification. In one study, machine learning tools aided clinicians in improving skin disease diagnosis accuracy by more than 33% compared to clinicians alone [28]. This promising result underscores the potential of machine learning algorithms in aiding dermatological care, offering new options for a more accurate and equitable future. However, the same study reported that the machine learning approach still struggled to accurately diagnose skin diseases in darker skin phototype patients based on the FSPC scale [28].

Classifying skin phototypes across diverse populations is challenging due to complex anatomical variability, differing tissue absorbance properties, and varied optical responses to broad-spectrum light. These challenges are further complicated by a mismatch between the salient optical properties of distinct skin structures, which are dynamic in response to broad-spectrum light spanning from UV to IR, in contrast to human vision, which operates in visible spectra [29]. At the cutaneous level, visual perception of skin color is affected by physical phenomena such as light scattering, absorption, and penetration depth within different skin layers (Figure 1b) [27]. It has been reported that 4–7% of a near-normal incident beam of light between 200–3000 nm is reflected away from the skin’s surface, or stratum corneum, regardless of skin type [27,30]. The remaining 93–96% of incident light is absorbed or refracted at the epidermis and dermis layers [31]. Melanin blocks UV light [32] and exists in two forms: pheomelanin (yellow-brown) and eumelanin (black-brown) [33,34]. Eumelanin is more abundant in the epidermis of individuals with darker skin types [35]. Despite efforts to correlate pigmentation, skin optical properties, and the FSPC scale, human color vision is most sensitive to blue, green, and red light spectra [23]. These narrower wavelengths do not fully account for the complex optical properties of human skin, which span the full photo spectrum. For example, melanosomes, a type of organelle in pigment cells, are reported to have scattering effects orders of magnitude higher than melanin’s absorbing effects across the Visible Light (VL) and Near-Infrared (NIR or Near-IR) spectra at 400–1,600 nm [35]. In contrast, melanin accounts for 50–75% of light absorption in the UV spectrum, leaving less light reflected in this spectrum among individuals with skin that contains high melanin concentration [36]. The complex optical properties of human skin, shortcomings of visual chromatic evaluations, and limitations in previous sensor-based work highlight an ongoing need for a more robust, objective skin phototype classification method with UV, Visible, and Near-IR spectra considerations. Such a method will provide a quantitative and repeatable framework for evaluating complex skin optical properties, thus yielding better diagnostic reliability and repeatability.

Here, we report an objective sensor-based skin optical properties classification approach that characterizes and classifies different skin phototypes based on optical similarities via a machine learning algorithm (K-means clustering) [37] across a 410940 nm spectral dataset for a culturally diverse subject pool. Historically, K-means classification models have been used as medical image classifiers for skin cancer detection and detection of human skin and gestures [38,39]. Based on computational simplicity and the ability to evaluate high-dimensional feature spaces, K-means clustering is a good candidate algorithm for evaluating and grouping sensor-derived broad-spectrum skin optical properties from diverse human populations. This proposed integrated sensor and algorithm approach serves not only to provide a framework for an objective, repeatable, and reliable skin type classification but also to further the understanding of the relationships between visual skin evaluations and underlying skin optical properties.

## 2. Materials and Methods

### 2.1. Participants

Thirty participants (16 females and 14 males) participated in this study. This cohort spanned an even distribution of the entire FSPC scale I–VI (five participants per category, Table 1). All enrolled participants were free of cardiopulmonary or respiratory disease. Table 1 summarizes participant demographic and anthropometric information. All research activities were approved by the University of Oregon Institutional Review Board, and written consent was obtained from all participants prior to the experiment.

### 2.2. Experimental Overview

**Figure 2:**
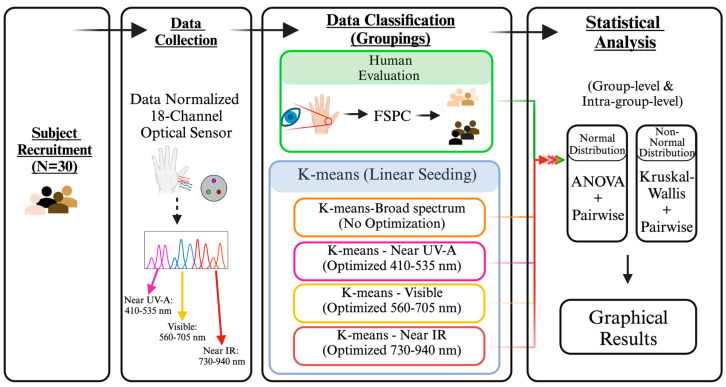
Block diagram of experimental procedures.

The experimental flow is illustrated in Figure 2. Participants first completed a questionnaire where they self-reported their age, biological sex, and ethnicity (Table 1). A trained research team member then evaluated the participants’ skin on the FSPC scale under standardized lighting conditions (lux: 967 lux, color temperature: 3442 K, confirmed by a spectrometer (Light Master IV, OPPLE, Shanghai, China). Skin was evaluated from the back of the hand between the thumb and index finger. This anatomic landmark was selected as the evaluation site for its typical skin uniformity, lack of hair, and unobtrusive access. Skin type was evaluated by comparing the skin color to the FSPC scale displayed on an OLED computer monitor presented in the same field of view. Left vs. right hand selection was randomized between participants.

After visual skin classification, participants underwent skin optical properties measurements with the spectral sensor (Section 2.3). The sensor was placed, contacting the participant’s skin at the same anatomic landmark as visual evaluations. Light pressure was applied to the sensor to isolate it from environmental light but not cause discomfort for the participant. Ten repeated samples were collected from each sensor (AS7261, 2, and 3), which measure irradiance at the Near-UV-A, Visible, and Near-IR spectra. In total, 180 data points (ten data points per channel across 18 channels) were collected for each participant; 16-bit data were transmitted to a PC via serial communication for subsequent processing (Section 2.4). Classification methods (FSPC vs. K-means) were then compared for their ability to differentiate skin phototypes (Section 2.5). The purpose of this study was to independently evaluate the FSPC and K-means methods for their ability to differentiate skin phototypes. The FSPC method was not considered the ground truth, and thus, direct comparisons for K-means vs. FSPC accuracy were not made.

### 2.3. Sensor Design

**Figure 3:**
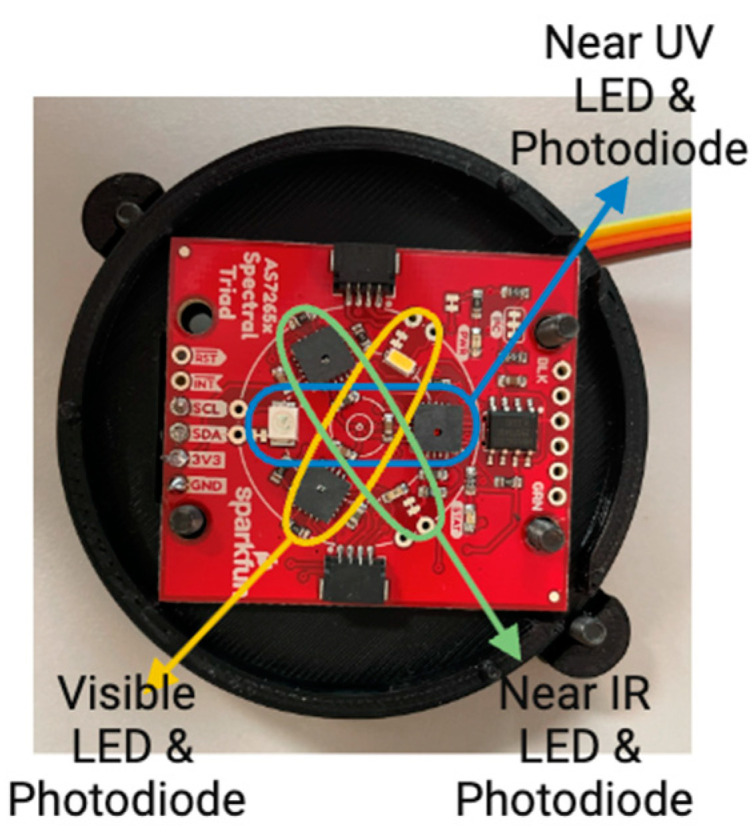
Sensor outside of packaging showing electronics, LEDs, and photodiodes.

The optical spectroscopy sensor (Figure 3) consists of spectrum-paired light-emitting diodes (LEDs) and photodiodes designed to probe the optical properties of skin. Three LEDs (Luxeon 3014, Lumileds, San Jose, CA, USA; VLMU3100, Vishay Semiconductors, Shelton, WA, USA; SIR19-21C/TR8, Everlight Electronics; New Taipei City, Taiwan) emit light spectra at 385–425 nm, 425–725 nm, and 780–950 nm bandwidths, respectively, spanning the Near-UV-A to Near-IR spectra. By combining three spectrophotometry sensors (AS72651, 2, and 3, AMS, Premstatten, Austria) with six bandpass filters each, the platform measures light resonance at 18 discrete wavelengths ranging from 390 nm to 960 nm with a sensitivity of 28.6 nW/cm^2^ of irradiance. The platform produces 16-bit digital irradiance values (range: 0–2^16^) at each wavelength via I2C communication for objective spectral characterization with data rates up to 400 kbit/s. Custom circuitry and firmware (C++, Visual Studio, Microsoft, Bellevue, WA, USA) on a microcontroller (WRL-15484, SparkFun, Niwot, CO, USA) controls the sensors and transmits data via serial connection to a PC for subsequent analysis. A 3D-printed (Prusa, Newark, DE, USA) Polylactic acid (PLA) sensor housing was designed to isolate the sensor from ambient light and allow measurements at specific spectra (i.e., Near-UV-A, Visible, or Near-IR) if desired.

### 2.4. Data Processing

Raw data from all participants were imported and processed in MATLAB R2023b (MathWorks. Inc, Natick, MA, USA). A broad description of the processing procedures is provided in Section K-Means Classification and Custom Seeding. An average irradiance value for each channel was calculated for each participant as the mean of the ten samples collected at each channel. Each channel’s data were then normalized to the maximum measurement observed in that channel, mapping each wavelength’s dataset on a 0 to 1 scale. The normalized participants’ measurements were then grouped based on the FSPC category, hereafter referred to as “human evaluation” in statistical comparisons or used as inputs to the K-means clustering algorithm (Section K-Means Classification and Custom Seeding) (Figure 2).

#### K-Means Classification and Custom Seeding

**Figure 4:**
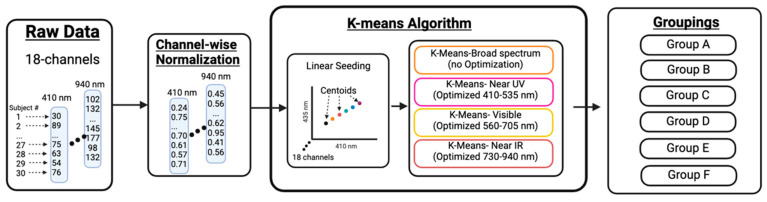
K-means classification workflow diagram.

The K-means clustering method (Figure 4) is a commonly used data classification approach that partitions data points into mutually exclusive clusters/groups (i.e., skin phototype) based on similarities in observed characteristics (i.e., irradiance measurements). This clustering method was selected for its simple mathematics, low computational power requirements, easily interpreted results, and comprehensive documentation in an effort to enhance clinical interpretation compared to more ambiguous black-box machine learning approaches. In this experiment, all 30 participants’ data were mapped in an 18-dimensional feature space, with each dimension representing the skin’s optical properties measured at a specific wavelength. Data were partitioned into *k* = 6 clusters based on the number of categories in the FSPC scale. The classification results arising from the K-means method do not follow the same ranked order logic as the FSPC. Instead, K-means provides a cluster of participants with similar optical properties across a particular bandwidth of interest. However, clusters with high irradiance values in a particular bandwidth may not be high in another, and thus, the ranked order logic should not be applied to K-means results.

The goal of this K-means classification approach is to find the six cluster centroid positions that best partition participants based on the least squares Euclidean distance of each data point to the centroid. The algorithm also seeks to maximize the Euclidean distance between centroids (i.e., maximize differentiation between groups). As such, initial centroid positioning can influence the ultimate classification results. In this study, randomly assigned centroid locations are not appropriate due to the nonnormal distribution of the data across 18 wavelengths. Moreover, the six centroids should not be initialized at the average irradiance value (or center of the domain) of each wavelength to avoid introducing wavelength weighting bias, which would skew classification results based on the wavelength with the highest irradiance value. To avoid these pitfalls, we utilized a custom centroid seed function whereby the centroids were initialized at evenly distributed positions along a linear function spanning the full irradiance range (minimum to maximum) observed at each wavelength. This approach provides a repeatable initial position for each centroid spanning the full range of irradiance values present in the dataset, reduces the chance of convergence at false local minima, and avoids introducing bias due to unequal irradiance values at different wavelengths.

To fully explore the classification capability of the K-means algorithm, four K-means analyses were performed. K-means_410–940_ (Broad-spectrum) used all 18 wavelengths as grouping criteria and categorized participants based on skin optical properties across the full bandwidth of light tested. K-means_410–535_ used six wavelengths ranging from 410–535 nm as grouping criteria and evaluated groupwise similarities across the Near-UV-A and lower visible spectra. Similarly, K-means_560–705_ and K-means_730–940_ used six wavelengths ranging from 560–705 nm and 730–940 nm, respectively, as grouping criteria to partition subjects across Visible and Near-IR spectra. By segmenting these spectra, we demonstrated this approach can be optimized for categorizing participant skin types based on specific bandwidths of interest and their associated clinical applications. For example, UV sensitivity with K-means_410–535_, skin chromatic appearances with K-means_560–705_, or therapeutic wavelength identification with K-means_730–940._ Additionally, this approach highlights that participants with similar skin types in a particular bandwidth are not necessarily similar at other bandwidths, demonstrating the need for customizability based on different clinical needs.

### 2.5. Statistical Analysis

In total, five classification grouping results (one human evaluation-classified dataset and four K-means-classified datasets) were individually investigated for their ability to classify skin type based on skin optical properties across the wavelengths tested. All statistical tests were performed in GraphPad 10 prism (GraphPad Software Inc., Boston, MA, USA). Each dataset was first tested for normality via the Shapiro-Wilk normality test (α = 0.05). If the normality assumption was met, the classification methods’ main effects of grouping on irradiance values were tested using one-way ANOVA analyses (α = 0.05) at each wavelength. If the normality assumption was violated, the nonparametric Kruskal–Wallis test (K-W) (α = 0.05) was used to evaluate the main effects. For all analyses, post-hoc pairwise comparisons were performed via Tukey’s Honest Significant Differences (HSD) tests (ANOVA) and Dunn’s method (K-W) with adjusted *p*-values to evaluate the resolution of the classification methods (i.e., the ability to differentiate between groups at each wavelength). Pairwise comparison *p*-values are reported in Appendix A.

An average Silhouette value [40] was also calculated for each cluster to quantify the quality of clusters generated by each K-means approach and FSPC evaluation. The Silhouette value is calculated as the difference between intra- and inter-cluster distances (average distance of a point to all other data points from the same cluster and average distance of this point to all other data points in the next nearest cluster, respectively) normalized to the maximum value of each. Silhouette values range from −1 to 1, with higher scores indicating better clustering quality.

## 3. Results and Discussion

### 3.1. Clustering Results 

Thirty subjects were classified based on four methods, and their respective grouping results were subsequently analyzed (Section 3.2 and Section 3.3). As shown in Table 2, the 30 subjects evenly covered the six skin phototypes of the FSPC scale based on human evaluation (*n* = 5 per FSPC group). However, based on skin optical data measured by the sensor, the K-means classification method grouped subjects nonuniformly across six categories. Moreover, in each of the K-means approaches, except K-means_410–535_, there were outlier groups in which one or more clusters contained only one participant. This occurs due to the lack of similarities with other subjects from the pool and may be explained by three causes: (1) the sample size is too small that no other subjects share similar optical properties with the outlier; and/or (2) *k* = 6 clusters is a suboptimal target for grouping (i.e., actual skin phototypes are more or less numerous than the FSPC); and/or (3) the evenly distributed channel-wise weight enlarged the differences between subjects that share similar optical properties. It is also important to note that there is no one-to-one matching relationship between the grouping results of human evaluation and K-means classification methods. It is important to note that for the purposes of this study, the FSPC scale was not regarded as the ground truth for classifying skin phototypes. Hence, there is no basis for comparison between methods for group size or accuracy. All classification results should be considered independent from one another. Groups with *n* = 1 were excluded from the statistical analysis.

The Silhouette values (SV) varied based on different clustering approaches (Table 3). Overall, the K-means_410–940_ resulted in the highest average SV (0.245 ± 0.358), with the FSPC Human Evaluation and K-means_730–940_ producing the lowest average SVs (−0.084 ± 0.387 and −0.088 ± 0.263). Clusters with SVs < 0.25 indicate low quality of data clustering (i.e., failure to identify and differentiate the distinctive optical features encoded within different skin types). In contrast, 0.25 < SV < 0.50 indicates weak-to-moderate quality, and SV > 0.50 indicates a cluster with high quality differentiation. Conditions with clusters with SV < 0.25 may indicate that *k* = 6 clusters is a suboptimal target. The K-means_410–940_ produced three adequate-quality clusters and one low-quality cluster, indicating that broadband optical data clustered into three groups may be viable. Future work should seek to further explore the optimal target number of clusters for maximizing the quality of data classification.

### 3.2. Group-Level Analysis

**Figure 5:**
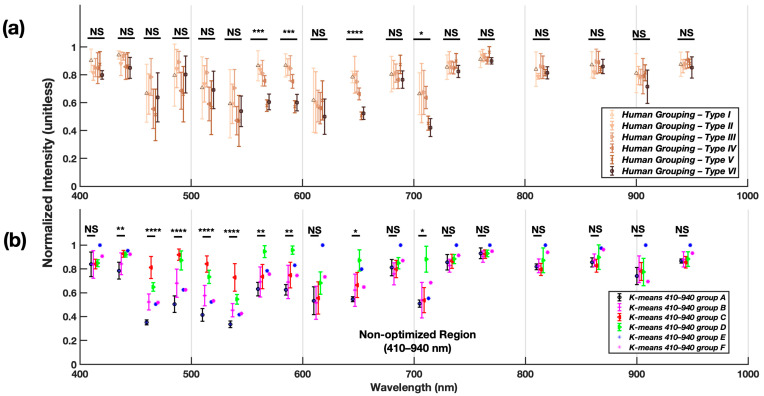
Normalized intensity of (**a**) human evaluation skin classification method vs. (**b**) K-means_410–940_ across a broad spectrum bandwidth; Significant main effects (α = 0.05) of the group on irradiance intensity are reported. NS: no statistical difference, *: *p* < 0.05, **: *p* < 0.01, ***: *p* < 0.001, ****: *p* < 0.0001. All group-level statistical values across different wavelengths can be found in Appendix A, Figure A1 and Figure A2.

As shown in Figure 5a, the human evaluation classification method resulted in statistically significant differences between skin types at only four wavelengths: 560 nm, 585 nm, 645 nm, and 705 nm, suggesting that humans rely primarily on these wavelengths when distinguishing skin types. However, the skin types differentiated at 705 nm failed to establish statistical significance when examined with pairwise comparison due to the lower adjusted *p*-value used (Section 3.3 and Appendix A). Moreover, the human classification method failed to establish significance from 410–535 nm, colors that span from blue to cyan. This observation aligns with previous research showing that human eyes are less sensitive to subtle differences from 390–500 nm spectra under the photopic condition [41] and more sensitive to distinguishing light in the yellow, green, and red spectra [41], which is also evident in the significant differences observed at 560 nm, 585 nm, and 645 nm in the human evaluation-classified condition (Figure 5a). This observation aligns with previous research showing that the trichromatic human photonic vision has a peak spectral sensitivity at approximately 555 nm, a combination wavelength from three types of short, middle, and long cone cells [42,43]. In summary, the chromatic assessment of the skin types via human evaluation only offers limited differentiation capability primarily constrained to a narrow bandwidth within the visible spectrum (560–645 nm) that leaves Near-UV-A and Near-IR spectrum unexplored.

While the results suggest that human evaluation-based classification relies primarily on visible light spectra for differentiating skin types, data from the K-means_410–940_ (Figure 5b) show that the Near-UV-A and lower visible wavelengths provide the most differentiating power across the bandwidth tested. When compared to the human evaluation condition, the K-means_410–940_ algorithm provided higher differentiation power, as shown in Figure 5b, showing statistical differences in an additional five wavelengths compared to human FSPC evaluations. More specifically, these additional wavelengths are typically found closer to the UV spectrum rather than the IR spectrum. This suggests that the actual optical properties of the skin may be encoded within the Near-UV-A and lower visible (435–585 nm) range, while the Near-IR spectrum (730–940 nm) may primarily encode information about body temperature, making it unsuitable for skin type classification.

**Figure 6:**
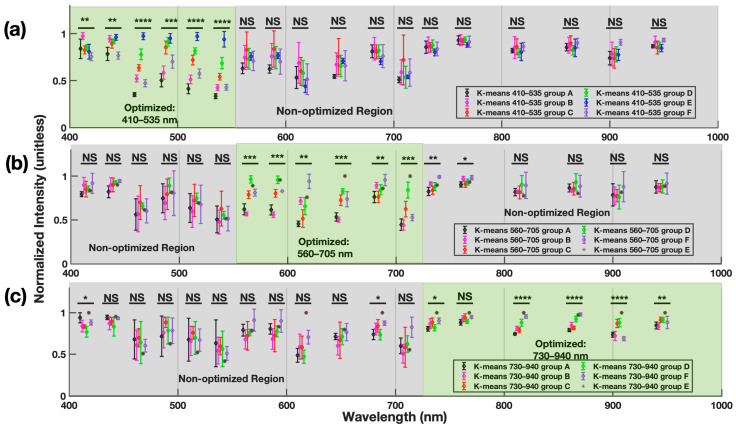
Normalized intensity of optimized (**a**) K-means_410–535_, (**b**) K-means_560–705_, and (**c**) K-means_730–940_ across a 410–940 nm bandwidth. Green shading denotes optimized bandwidths in the K-means classification approach, whereas grey shading denotes neglected bandwidths. Significant main effects (α = 0.05) of the group on irradiance intensity are reported. NS: no statistical differences, *: *p* < 0.05, **: *p* < 0.01, ***: *p* < 0.001, ****: *p* < 0.0001. All group-level statistical values across different wavelengths can be found in Appendix A, Figure A3, Figure A4 and Figure A5.

Figure 6 shows the optimized K-means classification results in which the algorithm was tuned to classify skin types based on the specific wavelengths of interest. Specifically, in Figure 6a, the K-means_410–535_ algorithm was applied with centroids’ seeding condition that focuses only on 410–535 nm and neglects other wavelengths. The results showed optimized dispersion in this spectrum specifically. This approach may have clinical significance for classifying skin phototypes for UV sensitivity rather than color (visible spectrum) or perfusion/temperature (IR spectrum). Similarly, desirable grouping results through visible spectrum optimization are shown in Figure 6b. In this approach, statistical differences were seen not only among the optimized wavelengths (560–705 nm) but also spanning further into part of the Near-IR (730–760 nm). In contrast to the clear and effective classification shown above in K-means_410–535_ (Near-UV-A) and K-means_560–705_ (Visible), the K-means_730–940_ method (Near-IR) shown in Figure 6c resulted in less dispersion between the classification groups. Nevertheless, significant main effects of group on intensity were observed in five of the six optimized wavelengths. In combination, bandwidth optimization of the K-means classification method can better differentiate various skin types through the wavelength spectrum from 410–705 nm. The less concentrated statistical differences of the IR optimization results might be due to the normalized intensity obtained through the Near-IR spectrum encoding body temperature instead of actual reflective skin optical properties.

In summary, the K-means classification methods provided better skin optical-based classification across the 410–705 nm spectrum (i.e., differentiation across different skin types) when compared to human evaluation classification based on the FSPC scale. Optimization of K-means classification based on the spectra of interest, such as Near-UV-A or Visible, offers unique opportunities to tailor skin type differentiation towards various medical applications. For example, the K-means method optimized on the Near-UV-A spectrum can be used to better assess the skin cancer risk among different groups of patients based on the skin’s optical properties instead of the skin color alone [39,44]. K-means optimized at the visible spectrum can be used to better match the cosmetic appearance of a skin graft [45]. The IR spectra-focused K-means classification can be utilized in identifying optimal therapeutic wavelength in photobiomodulation [9].

### 3.3. Intra-Group Analysis

**Figure 7:**
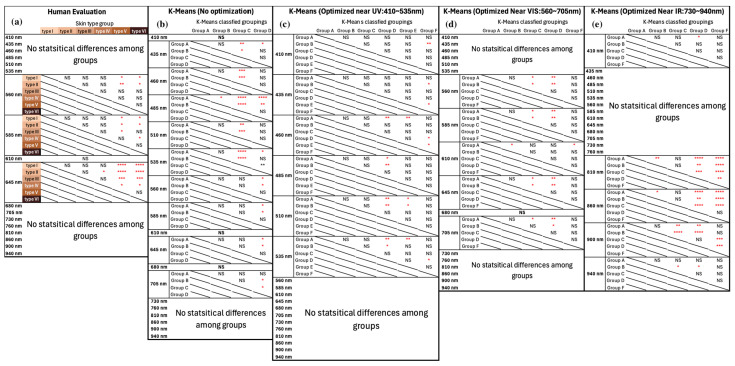
Table of statistical differences for intra-grouping pairwise comparison results at various wavelengths under different classification methods. (**a**) human FSPC classification method, (**b**) K-means_410–940_, (**c**) K-means_410–535_, (**d**) K-means_560–705_, (**e**) K-means_730–940_. Colors in (**a**) represent Fitzpatrick skin type scales I–VI. NS: no statistical difference, *: *p* < 0.05, **: *p* < 0.01, ***: *p* < 0.001, ****: *p* < 0.0001. All statistical tests excluded single-participant groupings. All intra-group level statistical values across different wavelengths can be found in Appendix A, Figure A1, Figure A2, Figure A3, Figure A4 and Figure A5.

Even though human eyes are most sensitive at 555 nm [42,43] and have shown the capability to differentiate between the extremes of the FSPC (e.g., I vs. VI), results from this study show that human visual evaluation struggled to differentiate between skin types that are more similar on the FSPC scale. As shown in Figure 7a, human FSPC evaluation showed significant differentiation between darker skin types (V and VI) and lighter skin types (I, II, and III) at 560 nm, 585 nm, and 645 nm. However, human eyes struggled to distinguish the finer differences among more similar skin types (i.e., I vs. II, II vs. III, etc.)

Compared to human evaluation, the non-optimized K-means classification (Figure 7b) displays more capability to differentiate skin types at 485 nm and 535 nm. Yet, it still struggled to distinguish different skin types, primarily in the Visible and Near-IR spectra. It is important to note that all K-Means classification methods, optimized or not, do not provide any one-to-one matching for their groupings to a specific FSPC category. Hence, no direct comparison between any two classification results can be conducted.

As described in Section 3.1 and shown in Table 2, K-means_410–535_ showed the same number of multi-participant clusters compared to human evaluation (*k = 6*). In contrast, all other K-means classifications resulted in at least one single-participant group. This observation suggests that K-means_410–535_ might share a similar classifying scheme as human evaluation whereas other K-means approaches do not. However, there is a discrepancy in the distribution of participants within those six groups, indicating that K-means_410–535_ and human evaluation may be using different grouping criteria. Previous research and the results of this study suggest that human evaluations rely specifically on a narrow bandwidth of light (560–645 nm) to differentiate skin phototypes, whereas K-means_410–535_ relies on a set of shorter wavelengths. When evaluated based on the highest percentage of possible differences among the skin type groups, the K-means_730–940_ method contains the most differentiable intra-grouping results (810–940 nm) among all K-means methods despite presenting an outlier group. However, this differentiation may be more representative of skin temperature, rather than phototype due to the role of temperature in IR wavelength absorption. Even though a comparison of direct grouping results is not achievable in this study, Figure 7 illustrates the resolution of each approach to classify skin phototypes.

In summary, K-means classification with optimization offers more customizable, quantifiable, and reproducible skin type classification based on skin optical measurements. However, this study is not without its limitations. For example, this study involved culturally diverse subject groups, but the sample size of the collection is limited and may not represent the full skin optics range present in the human population. Moreover, the sensors used to measure skin optics are not uniformly sensitive across the bandwidth tested. The Near-UV-A and lower visible spectrum wavelengths exhibited greater overall signal power than the other wavelengths tested, which could skew the weightings of these data in the clustering algorithms. Normalization procedures were implemented on each wavelength to reduce the weighting effects. Nevertheless, some effect of this weighting may have persisted in the final dataset, possibly masking finer differences between subjects difficult to detect due to the low signal-to-noise ratio for the channels with lower intensity. A related limitation is that K-means applies the same weighting factor to each wavelength and thus may not fully capture the varying skin optical properties at different wavelengths that drive skin type differentiation. The algorithm itself is also sensitive to the presence of outliers, the initial location of the centroids, and the uncertainty of optimal cluster numbers. Lastly, the 18 wavelengths sampled across 390–960 nm were not equally distributed (i.e., sampling intervals were non-uniform between wavelengths). These peak sensitivities are based on the bandpass filters designed by the manufacturer of the photodiodes selected for this study. It is possible that different sampling intervals or higher wavelength resolution could affect the results of this study.

Future work should seek to verify these results with broadened light spectra. Due to hardware limitations and participant safety, this study only utilized light in the Near-UV-A spectrum. This light may not share the same optical characteristics of light in the UV-B and UV-A spectra, which hold more clinical relevance for sunburn sensitivity, skin cancer risk, etc. Future work should also seek to verify these results with an increased sample size. A larger dataset would be advantageous for broadening the skin types observed and achieving a normal distribution in the dataset, allowing the use of ANOVA comparisons rather than the less statistically powerful Kruskal–Wallis, as was sometimes required for the present dataset. These factors will also help reduce the chances of single-participant groups in the results. Future work could also explore the use of more complex centroid seed functions (e.g., nonlinear), which might achieve better grouping results by narrowing the search space. Lastly, the results of this study suggest that *k* = 6 may not be the optimal target number of clusters. Future studies could benefit by including techniques such as the elbow method to investigate optimal cluster numbers.

## 4. Conclusions

This study presented empirical evidence showing that the combined sensor and K-means classification approach can be utilized for skin phototype classification. This method provides advantages over the conventionally used Fitzpatrick Skin Phototype Classification, including better differentiation power and the ability to optimize differentiation at specific bandwidths of interest. The K-means classification methods showed quantifiable and reproducible grouping results that can be segmentally applied to the 410–940 nm spectrum with various medical application focuses.

## Figures and Tables

**Table 1:** Summary table of participant demographic and anthropometric information.

FSPC Scale	Numbers of Participants	Age (Years) (Mean ± SD)	Biological Sex	Self-Identify Ethnicity
Type I	5	23.4 ± 5.78	M: 2 F: 3	White: 5
Type II	5	24.7 ± 6.00	M: 3 F: 2	Asian: 2 Hispanic: 1 White: 2
Type III	5	23.0 ± 4.41	M: 2 F: 3	Asian: 5
Type IV	5	22.4 ± 3.65	M: 3 F: 2	Asian: 1 Black: 4
Type V	5	23.9 ± 4.99	M: 4 F: 1	Asian: 1 Black: 4
Type VI	5	23.8 ± 5.05	M: 0 F: 5	Black: 5

SD: Standard deviation.

**Table 2:** Summary table of grouping results for human evaluation and K-means classification.

Human Evaluation		K-means Classification				
Skin Types	Subject Counts	Clusters	Subject Counts			
			Broad-Spectrum	Near-UV-A	Visible	Near-IR
Type I	5	Group A	3	3	7	4
Type II	5	Group B	9	5	4	11
Type III	5	Group C	12	6	13	6
Type IV	5	Group D	4	10	3	5
Type V	5	Group E	1	2	1	1
Type VI	5	Group F	1	4	2	3

**Table 3:** Summary of Silhouette values for the human evaluation and K-means approaches.

Silhouette Values			
	Cluster Type	Cluster Silhouette Value Mean ± SD	Method Silhouette Value Mean ± SD
**FSPC** **(Human** **Evaluation)**	**Type I**	−0.540 ± 0.080	−0.084 ± 0.387
	**Type II**	−0.374 ± 0.180	
	**Type III**	0.197 ± 0.243	
	**Type IV**	0.117 ± 0.432	
	**Type V**	−0.090 ± 0.280	
	**Type VI**	0.189 ± 0.344	
**K-means_410–940_** **(Broad spectrum)**	**Group A**	0.547 ± 0.131	0.245 ± 0.358
	**Group B**	−0.212 ± 0.162	
	**Group C**	0.438 ± 0.178	
	**Group D**	0.488 ± 0.137	
	Group E *	—	
	Group F *	—	
**K-means_410–535_** **(Near-UV-A)**	**Group A**	0.561 ± 0.073	0.159 ± 0.321
	**Group B**	−0.069 ± 0.202	
	**Group C**	−0.135 ± 0.321	
	**Group D**	0.251 ± 0.190	
	**Group E**	0.621 ± 0.127	
	**Group F**	0.119 ± 0.248	
**K-means_560–705_** **(Visible)**	**Group A**	0.179 ± 0.148	0.040 ± 0.214
	**Group B**	0.169 ± 0.212	
	**Group C**	−0.039 ± 0.183	
	**Group D**	−0.007 ± 0.298	
	Group E *	—	
	**Group F**	−0.124 ± 0.306	
**K-means_730–940_** **(Near-IR)**	**Group A**	−0.237 ± 0.075	−0.088 ± 0.263
	**Group B**	−0.062 ± 0.212	
	**Group C**	−0.204 ± 0.266	
	**Group D**	−0.100 ± 0.191	
	Group E *	—	
	**Group F**	0.264 ± 0.461	

* Cluster contains only one subject; thus, a Silhouette value cannot be calculated.

**Figure A1:**
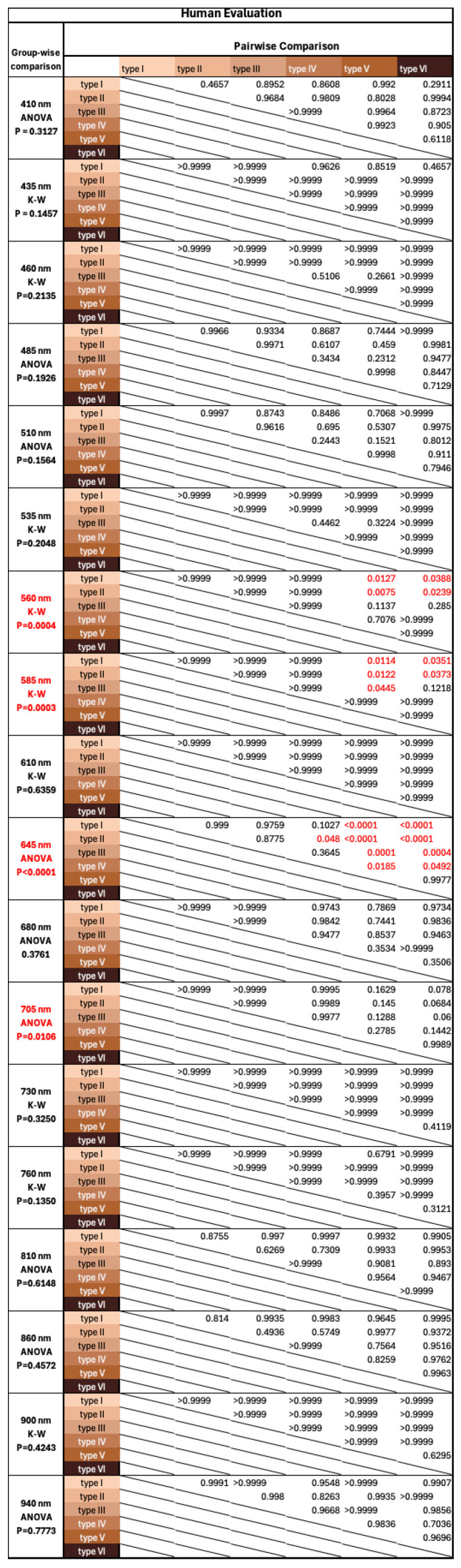
Groupwise (leftmost column) and pairwise (columns 2–8) comparison analysis results with *p*-value listed of human evaluation grouping results. (K–W: Kruskal–Wallis test). Red font denotes statistical significance.

**Figure A2:**
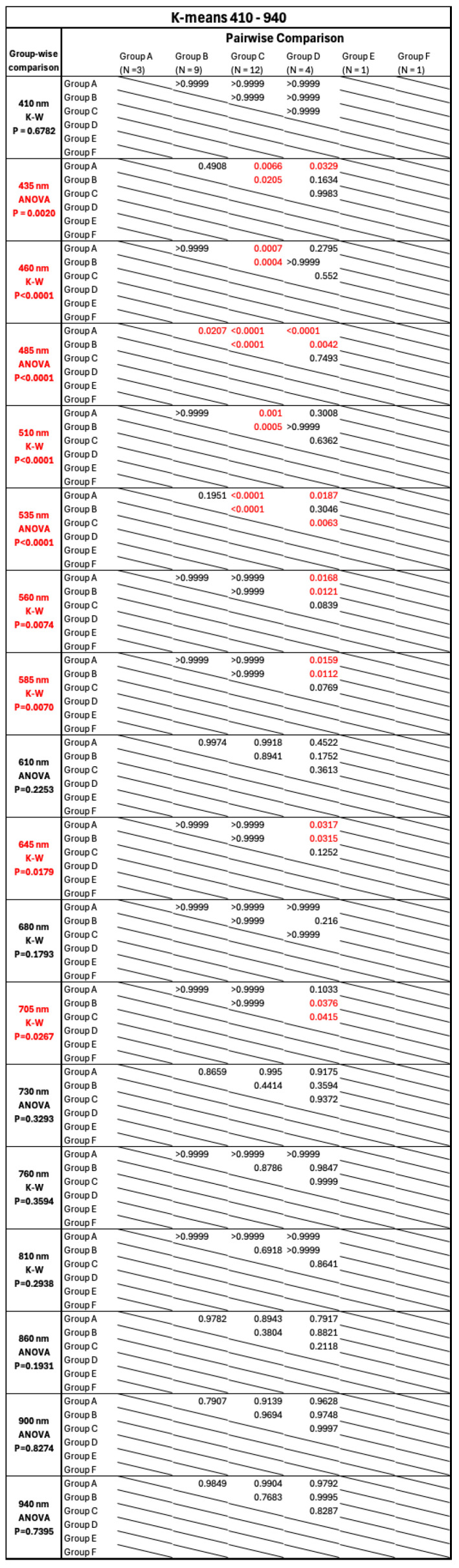
Groupwise (leftmost column) and pairwise (columns 2–8) comparison analysis results with *p*-value listed of K-means_410–940_ grouping results. (K–W: Kruskal–Wallis test). Red font denotes statistical significance.

**Figure A3:**
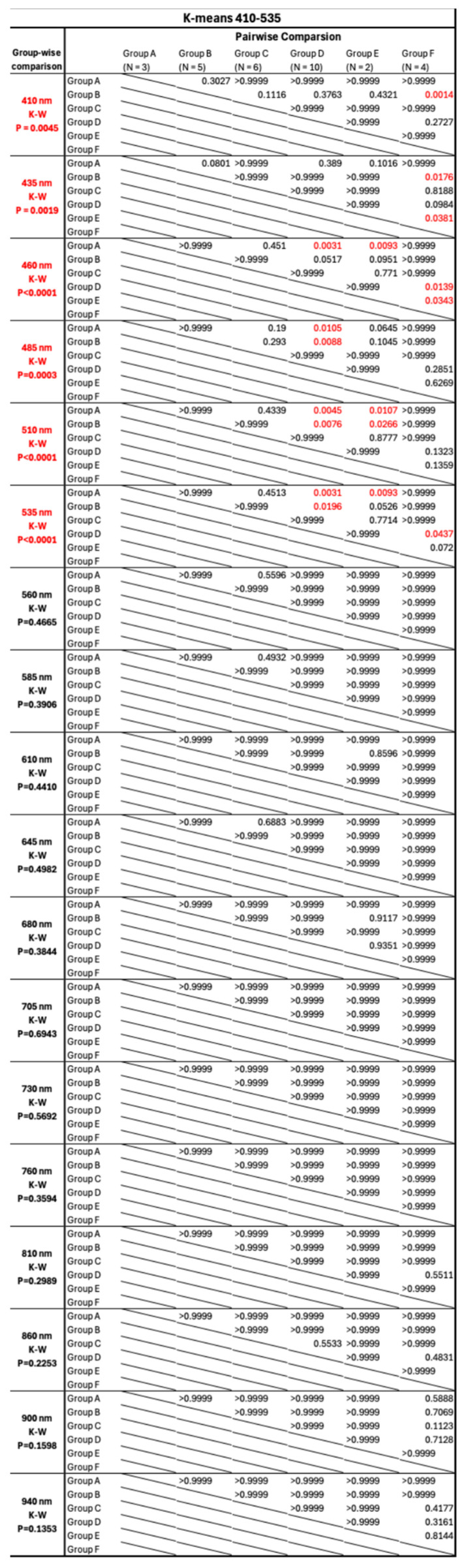
Groupwise (leftmost column) and pairwise (columns 2–8) comparison analysis results with *p*-value listed of K-means_410–535_ grouping results. (K–W: Kruskal–Wallis test). Red font denotes statistical significance.

**Figure A4:**
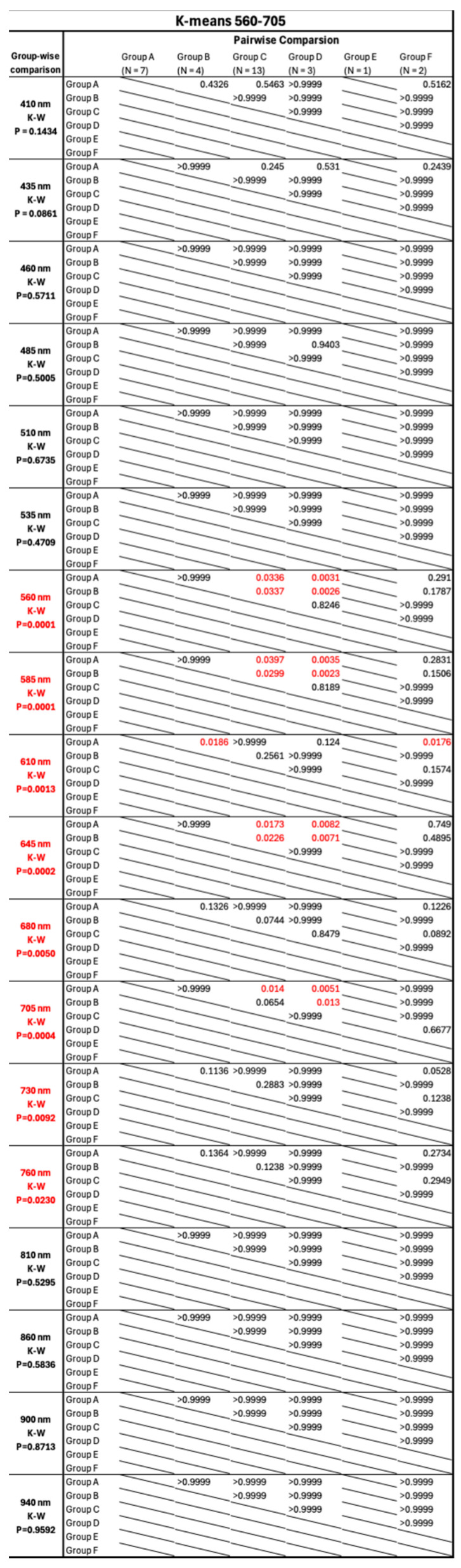
Groupwise (leftmost column) and pairwise (columns 2–8) comparison analysis results with *p*-value listed of K-means_560–705_ grouping results. (K–W: Kruskal–Wallis test). Red font denotes statistical significance.

**Figure A5:**
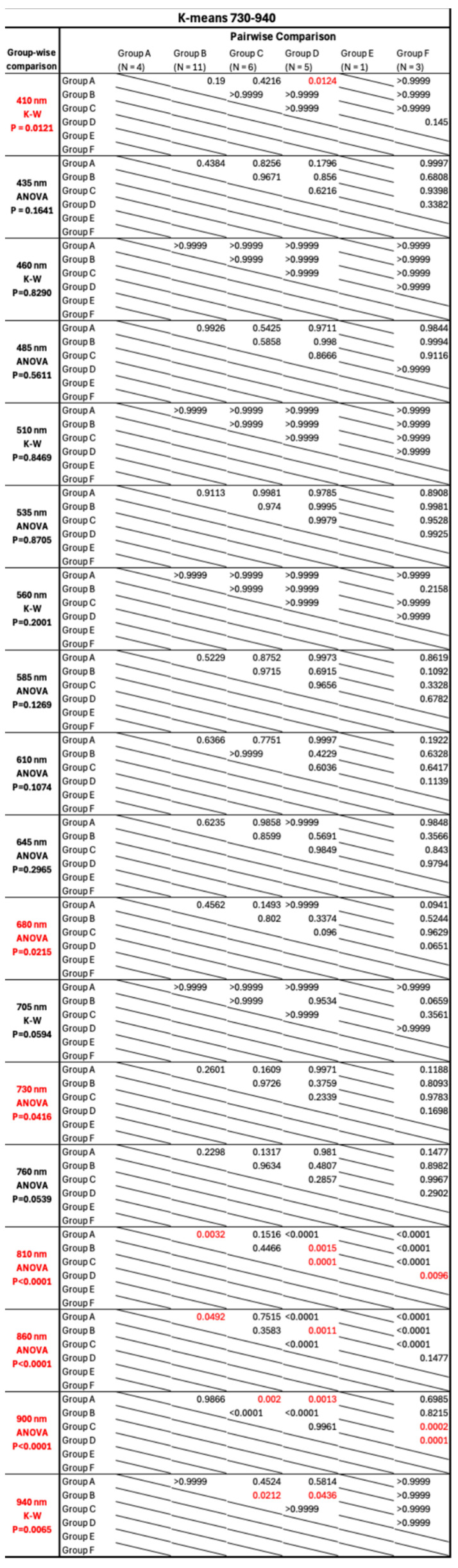
Groupwise (leftmost column) and pairwise (columns 2–8) comparison analysis results with *p*-value listed of K-means_730–940_ grouping results. (K–W: Kruskal–Wallis test). Red font denotes statistical significance.

## Data Availability

This study’s raw data and statistical analysis can be provided upon reasonable request to the authors and in compliance with the NIH guidelines for data sharing.
